# The Aims of Life

**Published:** 1854-11

**Authors:** 


### THE AIMS OF LIFE.

It is as true as truth, that the duties of life are more than life itself. To live, comprehends far more than the mere eating, drinking, and sleeping of every day existence. Gifted, as man is, with an active, inquiring mind; endowed with intellectual and moral powers; with a heart sensitive and alive to pleasure and to sorrow; with an immortal soul and an after destiny;—the ends and aims of his existence are certainly as lofty as the skies, and far above the little plans and schemes of life and enjoyment, which now so much occupy and engross the thoughts of mortals. Shakespeare speaks of "the touch of nature" which makes all men "kin"—uniting them together by a common bond of brotherhood. So to live as to benefit the human race, rather than to act as a bane and an injury, seems the duty of every one.

There is no doubt that an act of charity or benevolence done to another, creates in the human bosom a melody sweeter than the divinest harmony, and brings a reward far more precious than the diamonds of the richest mines, or the wealth of the most golden mountains. And in pursuing the journey of life—a rugged and thorny pathway, truly, with few flowers to beautify and adorn it—it is well to remember that all have cares and troubles and sorrows, which a little kindness may alleviate if not remove. It is well to remember that

> "Much of care
> Every human heart must bear,"

and that true benevolence consists not alone in good wishes, but in active deeds. The consciousness of having made one heart happier during the day, might well serve as a reward for the performance of a good action. The promptings of a benevolent nature are pure and lofty, and shed a tinge of tranquil happiness over the life of him who possesses it. As the morning dew arises to heaven rich with the fragrance of the flowers it has refreshed and brightened, so true benevolence ascends to the skies, laden with the blessings of all those upon whom it has shed one little ray of warm and genial sunshine.—Anon.

